# Niemann-Pick Disease Type A: A Rare Disease With a Fatal Outcome

**DOI:** 10.7759/cureus.21955

**Published:** 2022-02-06

**Authors:** Khadija Qureshi, Zahraa Ghasan Abdulmajeed, Shoaib Saleem, Javera Tariq, Mashhood Iqbal

**Affiliations:** 1 Internal Medicine, Bucks County Kidney Specialists, Langhorne, USA; 2 General Physician, Al-Mustansiriyah University, College of Medicine, Baghdad, IRQ; 3 Internal Medicine, Mayo Hospital, Lahore, PAK; 4 Pathology (Hematology), Pakistan Institute of Medical Sciences, Islamabad, PAK

**Keywords:** pancytopenia, rare autosomal recessive disorder, foamy histiocytes, lysosomal storage disorder, niemann-pick type a

## Abstract

Niemann-Pick disease (NPD) type A is a fatal autosomal recessive lysosomal storage disorder. This rare condition impairs the metabolization of lipids, leading to their accumulation within the cells. Consequently, it causes growth retardation, pancytopenia, and cellular malfunctioning in various organ systems, including ocular, hepatic, pulmonary, brain, and neuronal tissues. Although rare, these patients present in both emergency and outpatient settings. Here, we report the case of a seven-month-old male infant who presented to the emergency department with continuous fever for one week, poor feeding, and failure to thrive. After a thorough history, examination, and laboratory workup, NPD type A was diagnosed. The patient received symptomatic therapy with the continuation of conservative management. In addition, the parents received detailed counseling regarding the genetics, progressive disease course, and prognosis of this condition.

## Introduction

Niemann-Pick disease (NPD) is a disorder of lipid metabolization and is categorized as a lysosomal storage disease. It refers to a group of inherited metabolic disorders in which excessive and harmful accumulation of lipids occurs in various organs, particularly the brain, spleen, liver, lungs, and marrow bone. It follows an autosomal recessive inheritance pattern and is caused by acid sphingomyelinase deficiency (ASMD), which results in the accumulation of lipids in lysosomes, mainly in macrophages. The deposition of these lipid-laden macrophages in various organs leads to hepatosplenomegaly, cytopenias, lung disease, ocular abnormalities, and neurologic symptoms, including ataxia, loss of muscle tone, spasticity, brain degeneration, and speech articulation problems. Other manifestations of the disease can include hepatosplenomegaly, feeding and swallowing difficulties, ocular paralysis, and learning problems. NPD is classified into three main forms: type A, type B (both referred to as ASMD), and type C. Type A, also known as the “infantile neurovisceral form,” is caused by missense mutations in the *sphingomyelin phosphodiesterase 1* (*SMPD1*) gene. NPD type A has extremely low acid sphingomyelinase (ASM) activity, resulting in neurological deficits and growth retardation. It usually proves fatal before three years of age [1].

## Case presentation

A male infant, seven months of age, was brought to the emergency department (ED) with the chief complaint of continuous fever for one week, poor feeding, and failure to gain weight. The fever was persistent and documented at 100.7°F without rigors or chills. Over-the-counter acetaminophen syrup temporarily brought the fever down. His medical history showed multiple hospitalizations for recurrent lung and abdominal infections within four months. Medical records also revealed a significant lag on the growth chart with failure to achieve many developmental milestones. The patient was unable to hold his neck and could not roll over or sit without support. The infant was the firstborn of a consanguineous marriage. He was born via spontaneous vaginal delivery at 39 weeks of gestation, weighing seven and a half pounds (lbs). The patient was up-to-date on vaccinations. No known food or drug allergies were reported, and the clinical history was not significant. General physical examination showed an irritable, emaciated baby with pale skin. His weight and height were recorded at nine pounds (lbs) and fifty-one inches, respectively. Vital signs showed blood pressure of 90/60 mmHg, pulse of 62 beats per minute, a temperature of 101°F, and oxygen saturation of 96%. Abdominal examination showed a distended abdomen with hepatosplenomegaly on palpation. The liver was enlarged two-finger-breadths below the right costal margin, measuring 10 cm. The spleen was palpable four-finger-breadth below the left costal margin, measuring 13 cm. Ocular examination showed wandering eye movements with an inability to maintain eye contact. Neurological examination showed hypotonicity in all limbs with bilateral ankle and knee hyporeflexia. The rest of the examination was insignificant. A complete laboratory workup was significant for pancytopenia. Thyroid function and liver function tests were within normal range. Complete blood count (CBC) showed hemoglobin of 7.8 g/dL, mean corpuscular volume of 88 fL, hematocrit of 30%, mean corpuscular hemoglobin of 25 pg, platelets of 110 × 10⁹/L, and white blood cells of 2.9 × 10⁹/L (Table 1).

**Table 1:** Blood cell counts.

Parameter	Finding
Hemoglobin	7.8 g/dL
Mean cell volume	88 fL
Hematocrit	30%
Mean corpuscular hemoglobin	110 × 10⁹/L
White blood cells	2.9 × 10⁹/L
Platelets	110 × 10⁹/L

**Figure 1:**
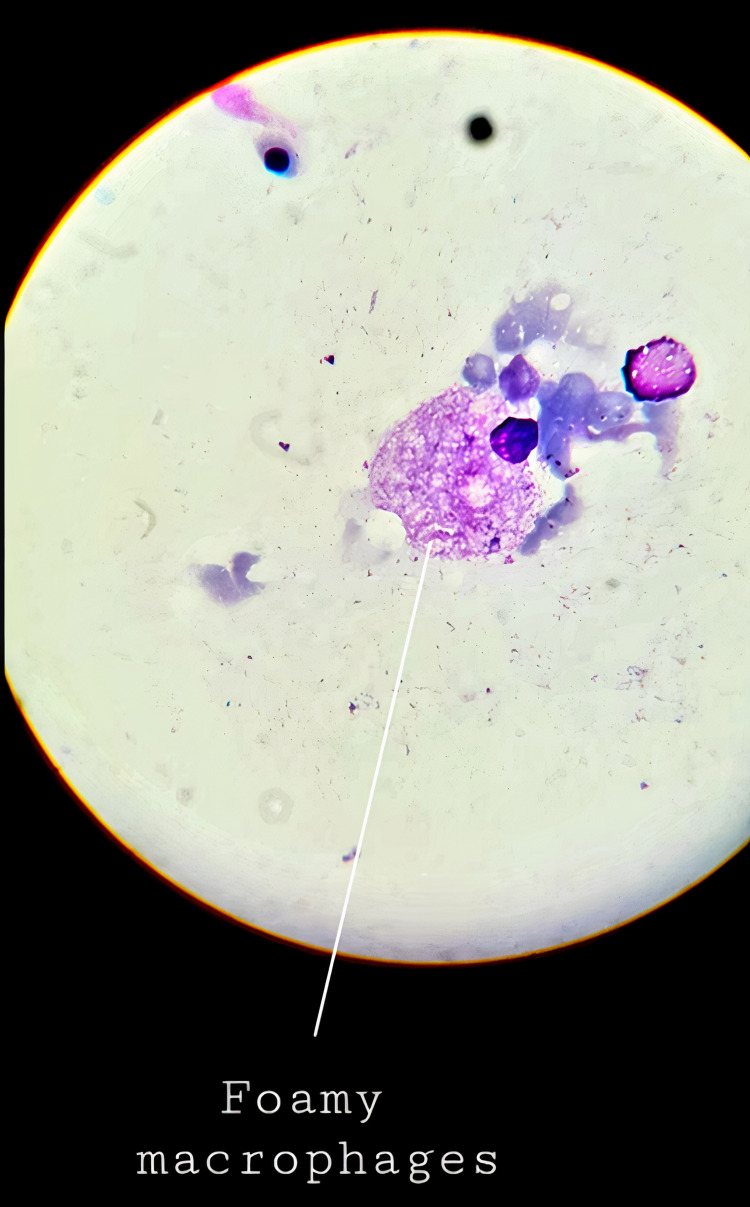
Foamy macrophages (Niemann-Pick cells) visualized on histopathological examination of bone marrow aspirate.

**Figure 2:**
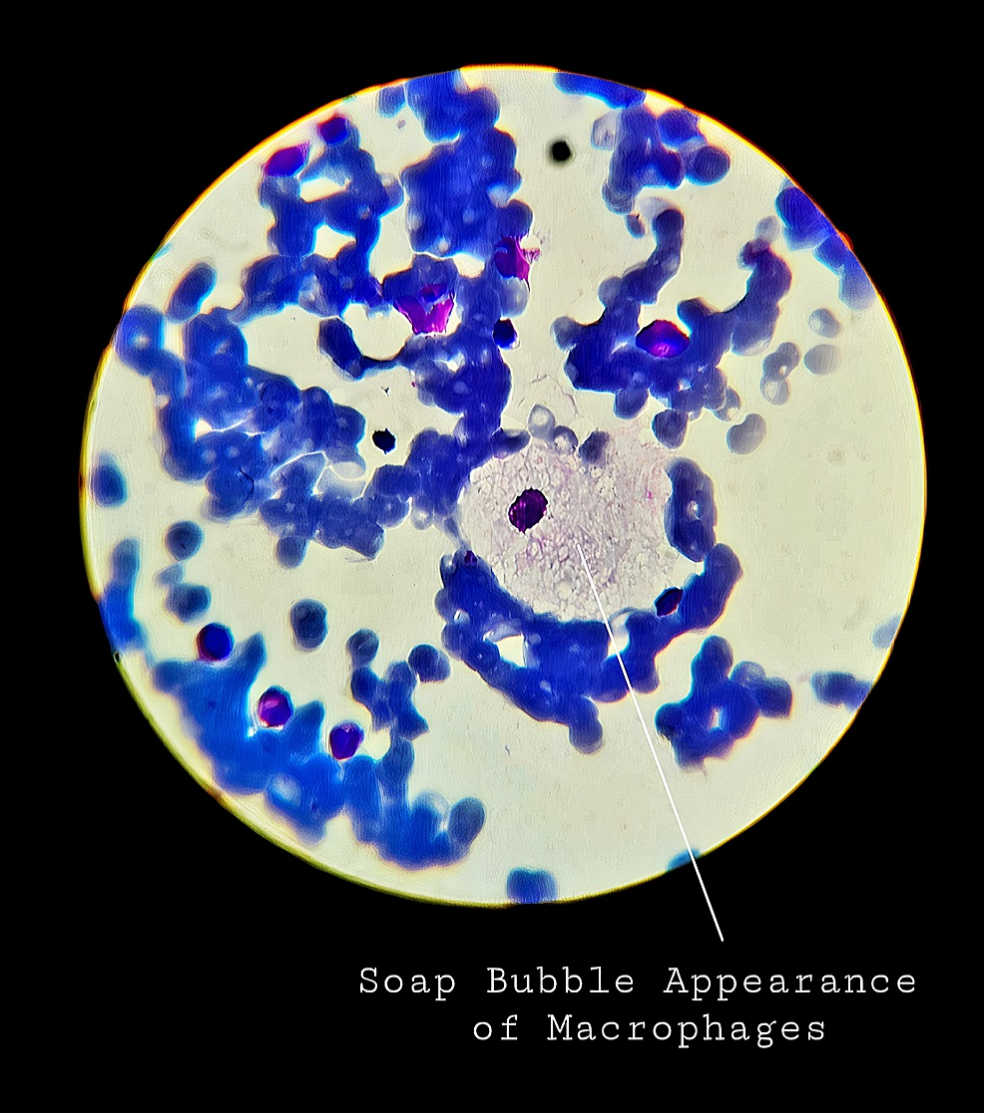
Foamy macrophages giving the classic “soap bubble appearance” on histopathological examination of bone marrow aspirate.

Abdominal ultrasonography revealed gross hepatosplenomegaly with hepatic size of 11 cm and spleen measuring 15 cm. Fundoscopy was performed which did not reveal any abnormalities. Additionally, a hemoglobin electrophoresis study did not yield any significant findings. To investigate the cause of his deranged blood results and imaging results, bone marrow (BM) aspiration was performed on the patient. The histopathology of BM aspirate revealed large foamy macrophages with sea-blue granules, staining positive on the acid-fast stain and negative on the periodic acid-Schiff (PAS) stain (Figures 1, 2). This finding pointed toward NPD type A as the most likely diagnosis.

After considering the presentation, medical history, blood results, and BM aspirate histopathology, the ASM enzyme assay was ordered, and markedly low activity of the enzyme was detected, thus confirming the diagnosis of NPD type A. The patient was managed symptomatically and supportive care was continued. The parents were extensively counseled regarding the genetics, clinicopathological nature, and prognosis of NPD type A. The patient was followed up for the next 10 months, during which he developed worsening neurological impairment with eventual areflexia and complete atonia. During the last three months of his life, he received feeding through a nasogastric tube. The patient underwent multiple hospitalizations secondary to recurrent gastrointestinal and pneumonic infections and died at 17 months after developing septic shock.

## Discussion

NPD type A is a rare and fatal lysosomal storage disease caused by an extremely low activity of the ASM enzyme, leading to the accumulation of sphingomyelin and its precursors in the cells. The incidence of NPD types A and B is 1 in 250,000 individuals with a high prevalence in Ashkenazi Jewish descent, affecting 1 in 40,000 individuals. NPD type A is an autosomal recessive disorder, meaning both alleles of the gene coding for the ASM enzyme are defective in affected individuals [1]. Autosomal recessive diseases typically affect both females and males equally. Additionally, consanguinity in a family affected by NPD often leads to children afflicted with the condition. Therefore, a thorough family medical history can prove crucial regarding clinical phenotypic patterns and genetic testing [2].

NPD type A is the infantile form with the most severe symptoms that eventually cause death, usually by the age of two to three years. NPD type B occurs later in childhood with a less severe disease course and better prognosis. NPD type C mainly affects adults, although the signs and symptoms can appear at any age, with asymptomatic hepatosplenomegaly being the most common finding. The infantile form or NPD type A starts manifesting during the initial months of life (usually around three months of age). This disease invariably affects all organs, and the presentation can be heterogeneous. Signs and symptoms include poor feeding, irritability, pancytopenias, hepatosplenomegaly, growth retardation, recurrent lung infections, recurrent mucosal bleeds, jaundice, interstitial lung disease, ataxia, and failure to thrive. By the age of one year, neurological symptoms such as the regression of developmental milestones and psychomotor retardation start appearing. Upon fundoscopic examination, all patients with NPD type A show the classical ophthalmologic finding known as the cherry-red spot [1]. The cherry-red spot refers to the red-tinted appearance of the macula surrounded by retinal opacification. In the case of NPD, the deposition of lipids in the retinal layers leads to the whitish appearance of the retina, making the macular region appear redder in contrast to the lipid-laden retinal cells [3].

To establish the diagnosis of NPD, a thorough history, detailed physical examination, fundoscopy, and laboratory workup, including CBC, liver function tests, basic metabolic profile, thyroid function tests, and lipid panel should be obtained. In addition, BM aspiration can help establish the diagnosis and show the extent of the disease. Infiltration of bone marrow by foamy macrophages and histiocytes appears as the classical “soap bubble appearance.” The foamy histiocytes, also called the Niemann-Pick cells, can be found in the reticuloendothelial systems of bone marrow, spleen, liver, and lymph nodes. These are round, large cells, up to 10-90 µm in diameter, and contain one or two peripheral nuclei. Their cytoplasm is filled with large lipid droplets, making it look like mesh or foam. The vacuoles of Niemann-Pick cells react strongly positive with Sudan black B, oil red O, and acid-fast stain, but negative or weakly positive with PAS stain [4].

In patients with suspected NPD, measuring the activity of the ASM enzyme in the leukocytes can confirm the diagnosis. Blood samples are taken from the patient to perform these enzyme tests. Low activity of the ASM enzyme is diagnostic of NPD. In addition, further disease evaluation and diagnostic confirmation can be achieved by performing molecular genetic analysis to detect mutations in the *SMPD1* gene, thus adding diagnostic precision [5]. In this case of our seven-month-old male patient presenting with fever and failure to thrive, detailed physical examination revealed numerous concerning findings, including an emaciated body habitus, palpable hepatosplenomegaly, defected ocular movements, hypotonicity, and hyporeflexia. The initial laboratory workup revealed pancytopenia, followed by a series of extensive blood tests and imaging studies. The combination of the clinical history and presentation, deranged blood cell counts, and abnormalities found on abdominal ultrasonography with no other identifiable cause led to performing BM aspiration. The histopathological examination of the BM aspirate revealed the typical Niemann cells or lipid-laden histiocytes, giving the classic foamy appearance. A diagnosis of NPD type A was made, and the low activity of ASM on enzyme assay confirmed the diagnosis. Conservative management and supportive therapy were the mainstays of the treatment plan. The care team followed up with the patient until the age of 17 months when a severe septic shock led to his demise.

NPD type A is considered incurable, whereas NPD type B shows a better prognosis. The patients are managed symptomatically, and treatment is mainly supportive. Children usually die from infections, fulminant hepatic failure, or progressive neurological loss. Other complications include atherosclerotic diseases and adverse coronary outcomes secondary to hyperlipidemia [6]. There is no effective treatment for NPD type A, although BM transplant is an option for variable improvement of these patients. Statins are used to lower the lipid levels with frequent monitoring of liver function and lipid panel [1]. Restriction of a fatty diet does not prevent the buildup of lipids in cells and tissues [7]. There have been trials of enzyme replacement and genetic therapies, but no promising treatments have been formulated yet. The prognosis remains poor, and the disease proves fatal within the first two to three years of life [1,7].

## Conclusions

NPD type A is a rare and fatal disorder that starts in early infancy and progressively worsens, leading to the patient’s death by two to three years of age. There are no effective treatments for NPD type A, and patients receive supportive care only. The prognostic improvement of NPD depends mainly on the success of the clinical trials striving to make enzyme replacement and genetic therapy possible for this incurable disease. Because it is an autosomal recessive condition, a significant yet modifiable risk factor for NPD is consanguinity. Therefore, physicians should encourage genetic counseling for their patients, especially patients from highly consanguineous communities such as those in the Middle East, Sub-Saharan Africa, South Asia, and Ashkenazi Jews.
